# mHealth Visual Discovery Dashboard

**DOI:** 10.1145/3123024.3123170

**Published:** 2017-09

**Authors:** Dezhi Fang, Fred Hohman, Peter Polack, Hillol Sarker, Minsuk Kahng, Moushumi Sharmin, Mustafa al'Absi, Duen Horng Chau

**Affiliations:** College of Computing, Georgia Tech; College of Computing, Georgia Tech; College of Computing, Georgia Tech; Dept. of Computer Science, University of Memphis; College of Computing, Georgia Tech, USA; Dept. of Computer Science, Western Washington University; University of Minnesota Medical School; College of Computing, Georgia Tech

**Keywords:** Visual analytics, health informatics, time series data, motif discovery, H.5.m [Information interfaces and presentation (e.g., HCI)]: Miscellaneous, J.3 [Computer Applications]: Life and Medical Sciences

## Abstract

We present Discovery Dashboard, a visual analytics system for exploring large volumes of time series data from mobile medical field studies. Discovery Dashboard offers interactive exploration tools and a data mining motif discovery algorithm to help researchers formulate hypotheses, discover trends and patterns, and ultimately gain a deeper understanding of their data. Discovery Dashboard emphasizes user freedom and flexibility during the data exploration process and enables researchers to do things previously challenging or impossible to do — in the web-browser and in real time. We demonstrate our system visualizing data from a mobile sensor study conducted at the University of Minnesota that included 52 participants who were trying to quit smoking.

## Introduction

When medical researchers conduct mobile sensor field studies, they often collect large amounts of time series data across many participants over prolonged periods of time. When incorporating data science techniques into the healthcare domain, making sense of the data collected from mobile health devices is essential for gaining actionable insights [3]. This volume of data (raw or pre-processed) can be overwhelming to a researcher seeking to gain such insights. For this reason, previous efforts such as TimeStitch often focus on high-level pattern summarization [2]. However, to test hypotheses and obtain a deep understanding of ones data, researchers need both low-level and high-level exploration tools for visualizing raw data, interactively inspecting it to formulate hypotheses, and discovering trends and patterns.

To address both low-level and high-level exploration, we present *Discovery Dashboard*: a visual analytics system that offers intuitive visualization of mobile sensor time series data, supports multiple interaction techniques for data and pattern exploration, and integrates a data mining algorithm for motif discovery.

## Mobile Sensor Dataset

We use data from a four day mobile sensor clinical study conducted at the University of Minnesota. The study aimed to uncover what causes relapse in cigarette smokers attempting to quit smoking. The research included a rich design to capture psychological, behavioral, biological, and physiological data related to stress, withdrawal symptoms, affect, and craving as well as lapse events for cigarette smokers attempting to quit [4]. From the 365MB dataset containing 52 participants, we visualize three time series (1 Hz) for each participant: inferred stress, physical activity, and heart rate, totaling 4.7M data points.

## Discovery Dashboard System and Design

**Figure 1:**
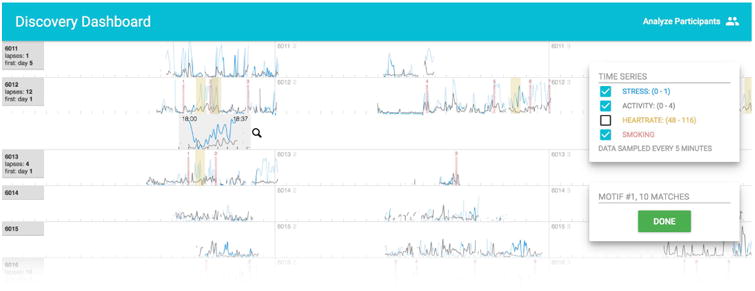
The Discovery Dashboard interface showing data from a mobile sensor study. Each row corresponds to one participant's data. A user-defined motif (for participant 6012) is selected, and the system automatically finds similar motifs across all participants and highlights them in yellow. This particular motif is a recurring pattern for participant 6012, often found near smoking lapses (vertical red dotted lines).

**Figure 2:**
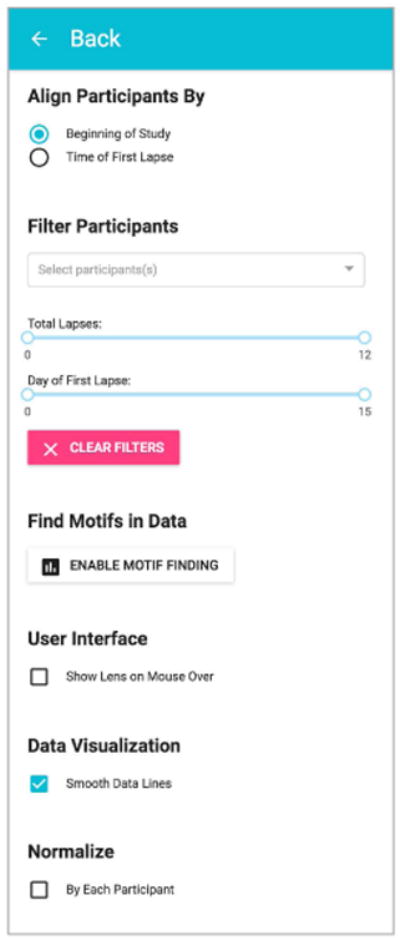
The Discovery Dashboard contains a number of options that are accessible from the “Analyze Particpants” button. Researchers can (1) align the data chronologically or by the first smoking lapse, (2) filter participants by name, number of lapses, and the day of their first lapse, and (3) search for time series motifs.

In Figure 1, Discovery Dashboard visualizes raw time series data of 52 participants, each represented by a single row, consisting of 24-hour blocks. Researchers can (1) align the time series by first smoking lapse, (2) filter participants by name, number of lapses, and the day of their first lapse, and (3) search for user-defined time series motifs (shown in Figure 2).

Discovery Dashboard is a web-based visualization system that can be run using any modern browser. However, using the web as a platform for making sense of large volume data presented interesting computational challenges. Below we describe some of our design decisions that enable the real time interactive experience in Discovery Dashboard.

### Scalability for Interactive Exploration

To support interactive exploration on data with high resolution, Discovery Dashboard needs to scale to large datasets; therefore we introduced multiple caching layers to achieve such scalability. Discovery Dashboard uses a relational database (SQLite) for storing the raw data and a key-value store (Redis) for caching resampled data and motif results. Time series data are pre-processed and manipulated with the Pandas package in Python and motif data are calculated using the Symbolic Aggregate approXimation (SAX) algorithm [1], a popular time series transformation method, written in Java. These services communicate via gRPC, a high performance remote procedure call (RPC) framework that transmits data using Google's Protocol Buffers. Calculated data are then transmitted to the client through WebSockets, transformed with D3.js, and rendered to the browser with React.js for maximum client performance.

### Motif Finding Algorithm

Finding motifs in time series data can be challenging, especially noisy data such as those collected from mobile sensor hardware. Discovery Dashboard uses the symbolic time series representation SAX algorithm [1] for its high performance when detecting latent patterns in noisy time series data. For example, in Figure 1, the zoomed in region under participant 6012 is used as motif query: similar patterns (highlighted in yellow) are found by SAX in the time series of participant 6012 and 6013, based on the patterns' similar “shapes” to the initial query motif, rather than their absolute temporal values.

### mHealth Time Series Alignment

Participants' time series data visualized in Figure 1 are aligned by the experiment start date, a natural alignment helpful for understanding the overall patterns across participants. However, aligning the visualization by other user-defined events, such as smoking lapses (vertical red dotted lines in Figure 1), can also help gain insights. For example, by aligning the data by first smoking lapses, we can more easily compare the different patterns that participants exhibited right before and after lapses.

## Demonstration Plan

We will engage our audience with the data described above by showing users a short live demo (refer to submitted video) that highlights the key visual analytics features and motif discovering tool. We invite our audience to try Discovery Dashboard, turn themselves into analysts, and make discoveries. We will also collect usability feedback.

For example, users can align data chronologically and by participants' first lapse, use filtering techniques to narrow the participant data into particular cohorts, and define motifs from zoomed selection in order to find trends in the dataset.

## Discussion and Ongoing Work

We are working to deploy Discovery Dashboard for health researchers and practitioners to evaluate the system's real-world usage. In addition, we are working to include Ecological Momentary Assessment (EMA) data into the Discovery Dashboard. EMA data are helpful for establishing qualitative contexts around the quantitative patterns that are measured using sensors. Exploring both sensor data and EMA data will allow us to gain additional insights that may not be offered by analyses separately performed for each data type.
